# Severe and Rare Case of Human *Dirofilaria repens* Infection with Pleural and Subcutaneous Manifestations, Slovenia

**DOI:** 10.3201/eid2812.221366

**Published:** 2022-12

**Authors:** Helena Biasizzo, Barbara Šoba, Frosina Ilovski, Matevž Harlander, Matej Lukin, Olga Blatnik, Matjaž Turel, Matevž Srpčič, Izidor Kern, Bojana Beović

**Affiliations:** University Medical Centre, Ljubljana, Slovenia (H. Biasizzo, F. Ilovski, M. Harlander, M. Lukin, M. Turel, M. Srpčič, B. Beović);; University of Ljubljana, Ljubljana (B. Šoba);; Institute of Oncology, Ljubljana (O. Blatnik);; University Clinic Golnik, Golnik, Slovenia (I. Kern)

**Keywords:** Dirofilaria repens, nematode, parasites, human, infection, pleural manifestations, subcutaneous manifestations, drug therapy, zoonoses, Slovenia

## Abstract

We report a case of human *Dirofilaria repens* infection in a woman in Slovenia who had concomitant pleural and subcutaneous manifestations of the infection. This case report illustrates the clinical course of a severe symptomatic parasitic infection that had multisystemic manifestations.

*Dirofilaria repens* is a filaria that causes infection primarily in dogs and other wild canids. The infection has been historically endemic in the Mediterranean countries. However, it has expanded to the rest of Europe in the past 2 decades, where it is considered an emerging infection (*1*).

Canids act as a reservoir for the parasite, where it can reach sexual maturity and produce microfilariae. Microfilariae are ingested by female mosquitoes, where they develop to infective larvae and are introduced to another definitive host during the blood meal of the vector (*2*). Humans can also acquire the infection, but are considered a dead-end host because microfilaremia in humans has only rarely been demonstrated (*3*,*4*).

Clinical symptoms of *D. repens* infection in humans are the consequence of the inflammatory reaction provoked by the migrating macrofilaria(e). The infection in humans is usually localized with only 1 parasite being found in most cases, and multiple parasites are rarely observed (*1*,*5*–*7*).

Depending on the anatomic location of the symptoms, *D. repens* infection has traditionally been divided into subcutaneous and ocular forms. The subcutaneous form is characterized by swelling and larva migrans‒like symptoms, whereas the ocular form shows visual disturbances caused by the migrating parasite (*1*,*7*,*8*). In addition, cases of other organ involvement have been reported, including testicles and lungs (*1*). Another, quite rare form is a pleural form, which manifests as an incidentally detected pleural lesion mimicking malignancy (*9*).

The course of this infection in humans is usually indolent, except for the ocular form, which can lead to loss of vision and, on rare occasions, involvement of vital organs, such as the central nervous system. Excision of macrofilaria is usually curative because only 1 parasite is usually present. However, in some cases treatment with antiparasitic drugs is needed (*10*). We report an unusual case in a patient who had pleural and subcutaneous *D. repens* clinical manifestations.

## The Study

**Figure 1:**
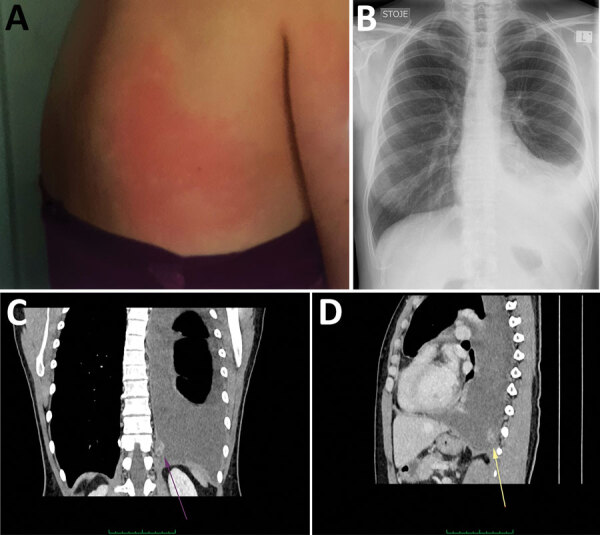
*Dirofilaria repens* infection with subcutaneous and pleural manifestations in a woman in Slovenia. A) Erythematous itchy skin lesion on the patient’s back measuring 15 cm x 15 cm (photograph taken by the patient). B) Frontal radiograph showing large left-sided pleural effusion. C, D) Contrast-enhanced computed tomography images showing large left-sided pleural effusion, uneven thickening of pleura, and a focal, heterogeneously enhancing soft tissue mass measuring 26 mm × 16 mm × 14 mm (arrows) in the posterior inferior part of the costal pleura in the coronal (C) and sagittal (D) plane.

A 40-year-old woman, a nonsmoker who had celiac disease but who was not receiving any regular medication and had no allergies, started having progressive dyspnea, dry cough, pain in the left hemithorax on inspiration, night sweats, and general malaise in September 2020. She reported no fever or weight loss. However, in addition, migratory, angioedematous skin changes measuring ≈15 cm × 15 cm started to appear on the upper trunk and axillary regions. Skin changes were present for 1–3 days and then disappeared (Figure 1, panel A). Her problems gradually intensified during the following months.

The patient sought medical attention in January 2021. Apart from decreased breath sounds on the left side, the clinical examination was unremarkable. Results for complete blood count, leukocyte differential counts, and C-reactive protein level were within reference ranges. A chest radiograph showed left-sided pleural effusion (Figure 1, panel B). Diagnostic thoracocentesis showed exudative pleurisy with lymphocytic predominance. There were no malignant cells, and results of microbiological examinations were negative.

Thorax and abdomen computed tomography scan showed a lesion adjacent to the left posterobasal pleura, pleural thickening suspicious for pleural carcinomatosis, left-sided pleural effusion, and reactive mediastinal and mesenteric lymph nodes (Figure 1, panels C, D).

A video-assisted thoracoscopic surgical excision of the pleural lesion was then performed. A total of 2,000 mL of serohemorrhagic pleural fluid was evacuated during the procedure.

**Figure 2:**
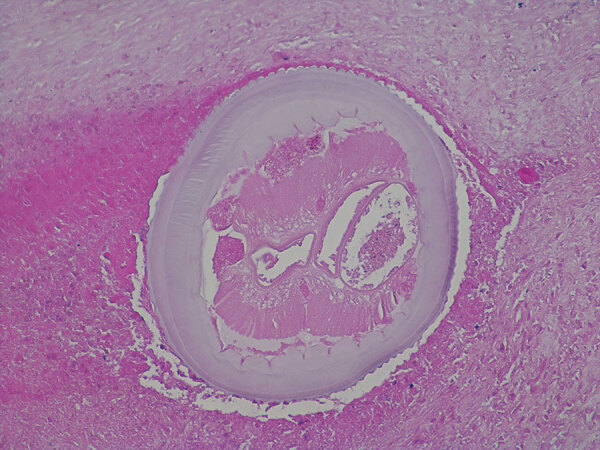
Photomicrograph of a cross-section of a *Dirofilaria repens* female in the background of necrosis in a sample from a woman in Slovenia. Image shows a multilayered cuticle with external ridges (feature that discriminates *D. repens* nematodes from other filariae infecting humans in the Mediterranean region); muscle cells, digestive tract and 2 uteri are well visible. The diameter at the widest point of the parasite is 0.6 mm. Hematoxylin and eosin stain; original magnification ×200.

**Figure 3:**
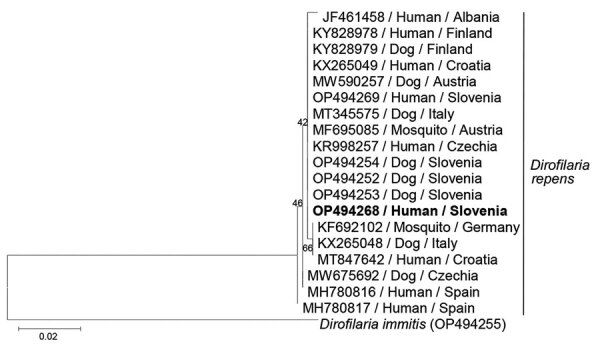
Phylogenetic tree of cytochrome c oxidase subunit 1 gene from a partial sequence of the *Dirofilaria repens* isolate from a woman in Slovenia (bold) and from closely related sequences of European *D. repens* isolates retrieved from GenBank. Tree was generated by the maximum-likelihood method based on a general time reversible model. A discrete gamma distribution was used to model evolutionary rate differences among sites (5 categories, parameter = 0.1737). Values on the branches are percentage bootstrap values using 1,000 replicates. GenBank accession number and host and geographic location are indicated at each node. DNA sequence of *D. immitis* parasite was used as an outgroup. Scale bar indicates nucleotide substitutions per site.

Histologic examination of the excised tissue showed necrotizing granulomas containing structures with a thick, laminated cuticle with external ridges, morphologically characteristic for the *Dirofilaria* spp. nematode (Figure 2). We extracted DNA from three 10 μm‒thick sections of formalin-fixed, paraffin-embedded tissue block by using a Deparaffinization Solution and a QIAamp DNA Mini Kit (all from QIAGEN, https://www.qiagen.com). *D. repens* infection was confirmed by using a species-specific, real-time PCR (qPCR), which amplified a 166-bp portion of the cytochrome c oxidase subunit 1 mitochondrial gene (*cox1*) (*11*). Sequencing and BLAST analysis (https://blast.ncbi.nlm.nih.gov/Blast.cgi) of a 689-bp fragment of *cox1* (*12*) showed 100% homology with several *D. repens* isolates from Europe (Figure 3) The sequence obtained has been deposited into GenBank (accession no. OP494268).

To detect microfilaremia, we extracted DNA from an EDTA‒whole blood sample of the patient by using the QIAamp DNA Mini Kit and performed a filarial qPCR, amplifying a portion of the 28S rRNA gene (*11*). The filarial qPCR result was negative.

Once the diagnosis was established, the patient was asked about possible risk factors for acquisition of the infection. She reported visiting the Istria region in Croatia in March 2020, where she had contact with stray dogs and was bitten by mosquitoes.

After excision of the pleural lesion and evacuation of the pleural fluid, respiratory symptoms of the patient slowly resolved. However, in March 2021, skin lesions persisted, and although not present initially, mild eosinophilia in peripheral blood (0.7 × 10^9^ cells/L) was detected. An ophthalmic examination was performed to exclude the ocular form and showed no abnormality. Treatment with ivermectin (200 μg/kg/d for 4 days) and doxycycline (100 mg 2×/d for 7 days) was initiated.

After treatment, the skin lesions completely disappeared and have not reappeared. The last computed tomography scan of the chest was performed in September 2021 and showed no pleural lesions or pleural effusion and the disappearance or regression of mediastinal and mesenteric lymph nodes. The patient has since remained without any problems.

## Conclusions

This case is noteworthy for several reasons. First, it illustrates human *D. repens* infection with pleural involvement, which is exceedingly rare. In our patient, the infection manifested not only with a pleural lesion but also with large pleural effusion, requiring evacuation of pleural fluid to relieve respiratory symptoms. In general, such clinical manifestations would be suspicious for malignancy, but this possibility was excluded by cytological and histological examinations.

Another filaria, *D. immitis*, which occurs sporadically in Slovenia and is endemic to neighboring Croatia, can also infect humans and cause predominantly pulmonary symptoms. Without detailed histological examination and molecular diagnosis, this clinical manifestation might be mistaken for *D. immitis* infection (*2*).

Second, unlike in most cases, it seems that our patient was infested with >2 macrofilariae, 1 located in the pleura and 1 located in the subcutaneous tissue of the upper body. Clinical manifestation with multiple parasites is rarely encountered in humans and might lead to microfilaremia in some exceptional cases (*3*–*6*). Because the filarial qPCR result of a whole blood sample with a detection limit of 1.5 × 10^−4^ microfilariae/mL of blood (*11*) was negative, microfilaremia was less probable for our patient.

Presentation with a subcutaneous nodule usually warrants surgical excision, which is diagnostic and curative. However, systemic treatment is needed if macrofilaria(e) cannot be surgically removed. Given the presence of numerous migratory erythematous lesions in our patient, antihelmintic therapy was prescribed.

The prevalence of *D. repens* infection among dogs in Slovenia is estimated to be 0.64%, whereas in some parts of Croatia it can be as high as 47.3% (*13*,*14*). Moreover, there have been case reports of human dirofilariasis in recent years in Slovenia (*13*,*15*). It can be assumed that the patient acquired the infection while visiting Croatia, although autochthonous infection cannot be ruled out.

This case illustrates an unusual manifestation of a *D. repens* nematode infection in a human. Clinicians should become familiar with possible clinical manifestations of this parasitosis, because we can expect an increased number of future cases.
